# Supplementary material for: Diet-Induced Gut Barrier Dysfunction Is Exacerbated in Mice Lacking Cannabinoid 1 Receptors in the Intestinal Epithelium
Source: Int J Mol Sci. 2022 Sep 11;23(18):10549. doi: 10.3390/ijms231810549 (PMC9504303; doi:10.3390/ijms231810549)
Supplement: Supplementary file 1 [file ijms-23-10549-s001.zip › ijms-1875298-supplementary.pdf]

Supplementary Table S1

	SD	WD
DGL Activity (nmol/mg/mL)	1.94 ± 0.25	1.25 ± 0.19<br>t(11) = 2.25 ; *p = *0.0461*
MGL Activity (nmol/mg/mL)	24.09 ± 1.048	23.65 ± 1.789<br>t(12) = 0.19; p = *0.8510*
2-AG (nmol/g)	24.078 ± 2.031	17.999 ± 1.582<br>t(13) = 2.39; *p = *0.0326*
2-DG (nmol/g)	5.646 ± 1.616	1.829 ± 0.4679<br>t(11) = 2.77; *p = 0.0184
2-LG (nmol/g)	181.787 ± 42.377	40.732 ± 11.360<br>t(12) = 3.66; **p = *0.0033*
2-OG (nmol/g)	97.678 ± 17.207	74.170 ± 17.339<br>t(12) = 0.94; p = *0.3656*
AEA (pmol/g)	10.04 ± 1.892	10.14 ± 1.879<br>t(9) = 0.03; p = *0.9710*
DHEA (pmol/g)	34.76 ± 3.677	35.65 ± 2.800<br>t(12) = 0.20; p = *0.8481*
OEA (pmol/g)	343.4 ± 57.93	156.7 ± 9.33<br>t(13) = 3.41; **p = *0.0047*

Supplementary Table S1 DGL activity, MGL activity, and lipid levels in the large intestinal epithelium of mice fed SD or WD. Statistical differences between SD and WD were determined via t-tests (two-tailed, unpaired); data displayed includes t-values, degrees of freedom, and p-values; * = $p < 0.05$, ** = $p < 0.01$. SD = standard diet, WD = western diet.

Supplementary Table S2

**Gene of interest**	**$intCB_1$+/+**	**$intCB_1$-/-**
Tjp-1 mRNA<br>(Relative Expression)	1.00 ± 0.30	0.89 ± 0.37<br>$t(10) = 0.19$; $p = 0.854$
Ocln mRNA<br>(Relative Expression)	1.00 ± 0.25	0.76 ± 0.13<br>$t(10) = 0.93$; $p = 0.372$
Cldn-1 mRNA<br>(Relative Expression)	1.00 ± 0.42	1.28 ± 0.26<br>$t(13) = 0.61$; $p = 0.551$
Cnr1 mRNA<br>(Relative Expression)	1.00 ± 0.27	0.36 ± 0.03<br>$t(11) = 2.54$; $^{*}p = 0.027$

Supplementary Table S2: Relative expression levels of mRNA relative to Hprt housekeeping gene in $intCB_1$+/+ control mice and $intCB_1$-/- mice. Details of the unpaired multiple t-tests analysis performed on RT-qPCR quantified genes in baseline $intCB_1$+/+ and $intCB_1$-/- mice. Data displayed includes t-ratios, degrees of freedom, and p-values; * = $p < 0.05$.

Supplementary Table S3

	+/+ SD vs -/- SD	+/+ SD vs +/+ WD	-/- SD vs -/- WD	+/+ WD vs -/- WD
Week 0 FITC-Dextran (μg/mL)	0.73 ± 0.14 vs. 0.58 ± 0.11<br>t(28) = 0.81; F = 1.45<br>n.s. *p* = 0.425	N/A	N/A	N/A
Week 2 FITC-Dextran (μg/mL)	0.66 ± 0.08 vs. 0.49 ± 0.04<br>t(25) = 0.897<br>n.s. *p* = 0.6124	0.66 ± 0.08 vs. 1.06 ± 0.17<br>t(25) = 2.286<br>n.s. *p* = 0.1185	0.49 ± 0.04 vs. 0.98 ± 0.13<br>t(25) = 2.692<br>n.s. *p* = 0.0609	1.06 ± 0.17 vs. 0.98 ± 0.13<br>t(25) = 0.458<br>n.s. *p* = 0.6509
Week 8 FITC-Dextran (μg/mL)	0.56 ± 0.05 vs. 0.58 ± 0.10<br>t(26) = 0.092<br>n.s. *p* = 0.9274	0.56 ± 0.05 vs. 1.28 ± 0.21<br>t(26) = 2.65<br>**p = 0.0396*	0.58 ± 0.10 vs. 2.45 ± 0.28<br>t(26) = 7.35<br>****p < 0.0001*	1.28 ± 0.21 vs. 2.45 ± 0.28<br>t(26) = 4.45<br>****p = 0.0006*
Epididymal Fat (mg)	0.62 ± 0.09 vs. 0.64 ± 0.12<br>t(53) = 0.093<br>n.s. *p* = 0.9261	0.62 ± 0.09 vs. 2.04 ± 0.21<br>t(53) = 6.73<br>****p < 0.0001*	0.64 ± 0.12 vs. 2.44 ± 0.14<br>t(53) = 8.41<br>****p < 0.0001*	2.04 ± 0.21 vs. 2.44 ± 0.14<br>t(53) =1.92<br>n.s. *p* = 0.1165
Large Intestine Length (cm)	7.63 ± 0.16 vs. 7.32 ± 0.16<br>t(53) = 1.56<br>n.s. *p* = 0.2357	7.63 ± 0.16 vs. 6.87 ± 0.14<br>t(53) = 3.95<br>***p = 0.0012*	7.32 ± 0.16 vs. 6.75 ± 0.08<br>t(53) = 2.93<br>**p = 0.0199*	6.87 ± 0.14 vs. 6.75 ± 0.08<br>t(53) = 0.608<br>n.s. *p* = 0.5458
Large Intestine Weight (mg)	189 ± 5.47 vs. 168 ± 10.86<br>t(42) = 1.65<br>n.s. *p* = 0.428	189 ± 5.47 vs. 166 ± 9.96<br>t(42) = 1.88<br>n.s. *p* = 0.342	168 ± 10.86 vs. 173 ± 7.60<br>t(42) = 0.35<br>n.s. *p* = 0.929	166 ± 9.96 vs. 173 ± 7.60<br>t(42) = 0.55<br>n.s. *p* = 0.929
Weight to Length Ratio (mg/cm)	24.9 ± 0.72 vs. 23.2 ± 1.88<br>t(42) = 0.87<br>n.s. *p* = 0.915	24.9 ± 0.72 vs. 24.6 ± 1.57<br>t(42) = 0.19<br>n.s. *p* = 0.944	23.2 ± 1.88 vs. 25.5 ± 1.19<br>t(42) = 1.15<br>n.s. *p* = 0.832	24.6 ± 1.57 vs. 25.5 ± 1.19<br>t(42) = 0.50<br>n.s. *p* = 0.944

Supplementary Table S3: Summary of data and t-tests performed on serum FITC-Dextran at baseline (Week 0) and the ordinary two-way ANOVA analysis of weeks two and eight serum FITC-Dextran, epididymal fat mass, large intestine length, weight, and weight to length ratio across diet or genotype. Holm-Sidak's multiple comparison test was performed as post-hoc analysis. Data displayed includes F-values, t-ratios, degrees of freedom, and p-values; * = *p*< 0.05, ** = *p*< 0.01, *** = *p*< 0.001. SD = standard diet, WD = western diet, n.s. = not significant.

Supplementary Table S4

Transcript of Interest	+/+ SD vs -/- SD (Welch's t-test)	+/+ SD vs +/+ WD (Welch's t-test)	-/- SD vs -/- WD (Welch's t-test)	+/+ WD vs -/- WD (Welch's t-test)
CXCL13	1.00 ± 0.30 vs. 0.86 ± 0.10<br>t(2) = 0.428; F = 9.00<br>n.s. *p* = 0.352	1.00 ± 0.30 vs. 1.56 ± 0.14<br>t(3) = 1.69; F = 4.96<br>n.s. *p* = 0.098	1.00 ± 0.12 vs. 1.55 ± 0.11<br>t(5) = 3.49; F = 1.67<br>***p = 0.008*	1.00 ± 0.09 vs. 0.86 ± 0.06<br>t(4) = 1.34; F = 1.08<br>n.s. p = 0.127
CXCR3	1.00 ± 0.21 vs. 0.68 ± 0.10<br>t(3) = 1.36; F = 4.90<br>n.s. *p* = 0.137	1.00 ± 0.21 vs. 0.83 ± 0.07<br>t(2) = 0.76; F = 10.3<br>n.s. *p* = 0.258	1.00 ± 0.14 vs. 2.08 ± 0.44<br>t(6) = 2.36; F = 19.1<br>**p = 0.029*	1.00 ± 0.08 vs. 1.71 ± 0.36<br>t(5) = 1.93; F = 40.1<br>n.s. *p* = 0.053
IFNGR1	1.00 ± 0.14 vs. 0.93 ± 0.06<br>t(3) = 0.49; F = 5.11<br>n.s. *p* = 0.330	1.00 ± 0.14 vs., 0.92 ± 0.04<br>t(2) = 0.56; F = 10.0<br>n.s. *p* = 0.311	1.00 ± 0.07 vs. 0.72 ± 0.07<br>t(6) = 2.91; F = 2.14<br>**p = 0.014*	1.00 ± 0.05 vs. 0.73 ± 0.07<br>t(7) = 3.23; F = 4.18<br>***p = 0.007*
ICAM1	1.00 ± 0.10 vs. 1.13 ± 0.18<br>t(3) = 0.65; F = 3.14<br>n.s. *p* = 0.281	1.00 ± 0.10 vs. 1.20 ± 0.10<br>t(4) = 1.44; F = 1.02<br>n.s. *p* = 0.112	1.00 ± 0.16 vs. 1.97 ± 0.30<br>t(7) = 2.90; F = 7.23<br>**p = 0.012*	1.00 ± 0.08 vs. 1.85 ± 0.28<br>t(6) = 2.95; F = 23.2<br>**p = 0.013*
HLA-DRA	1.00 ± 0.26 vs. 1.11 ± 0.44<br>t(3) = 0.22; F = 2.97<br>n.s. *p* = 0.420	1.00 ± 0.26 vs. 0.99 ± 0.23<br>t(4) = 0.04; F = 1.25<br>n.s. *p* = 0.486	1.00 ± 0.40 vs. 3.11 ± 0.81<br>t(7) = 2.35; F = 8.33<br>**p* = 0.026	1.00 ± 0.23 vs. 3.51 ± 0.91<br>t(6) = 2.67; F = 30.9<br>**p = 0.020*
CD247	1.00 ± 0.23 vs. 0.98 ± 0.26<br>t(4) = 0.05; F = 1.19<br>n.s. *p* = 0.483	1.00 ± 0.23 vs. 1.27 ± 0.07<br>t(2) = 1.12; F = 12.5<br>n.s. *p* = 0.183	1.00 ± 0.26 vs. 3.20 ± 0.72<br>t(6) = 2.88; F = 15.4<br>**p = 0.014*	1.00 ± 0.05 vs. 2.48 ± 0.56<br>t(5) = 2.64; F = 229<br>**p = 0.023*
HLA-DQA1	1.00 ± 0.14 vs. 0.81 ± 0.03<br>t(2) = 1.32; F = 21.6<br>n.s. *p* = 0.154	1.00 ± 0.14 vs. 0.82 ± 0.03<br>t(2) = 1.23; F = 29.2<br>n.s. p = 0.169	1.00 ± 0.04 vs. 1.24 ± 0.09<br>t(7) = 2.57; F = 10.3<br>**p = 0.020*	1.00 ± 0.03 vs. 1.22 ± 0.08<br>t(6) = 2.42; F = 13.9<br>**p = 0.025*
CXCR6	1.00 ± 0.18 vs. 0.84 ± 0.19<br>t(4) = 0.64; F = 1.10<br>n.s. *p* = 0.278	1.00 ± 0.18 vs. 1.18 ± 0.03<br>t(2) = 0.97; F = 32.8<br>n.s. *p* = 0.214	1.00 ± 0.22 vs. 1.82 ± 0.14<br>t(4) = 3.14; F = 1.29<br>**p* = 0.020	1.00 ± 0.03 vs. 1.30 ± 0.10<br>t(6) = 2.90; F = 28.0<br>**p = 0.015*
CXCR5	1.00 ± 0.26 vs. 0.89 ± 0.09<br>t(2) = 0.40; F = 8.92<br>n.s. *p* = 0.360	1.00 ± 0.26 vs. 1.27 ± 0.24<br>t(4) = 0.87; F = 1.24<br>n.s. *p* = 0.243	1.00 ± 0.10 vs. 2.07 ± 0.29<br>t(6) = 3.52; F = 17.1<br>***p = 0.006*	1.00 ± 0.19 vs. 1.45 ± 0.20<br>t(6) = 1.65; F = 2.38<br>n.s. *p* = 0.075
IL1A	1.00 ± 0.25 vs. 1.12 ± 0.16<br>t(3) = 0.41; F = 2.44<br>n.s. p = 0.352	1.00 ± 0.25 vs. 1.06 ± 0.01<br>t(2) = 0.22; F = 776<br>n.s. *p* = 0.422	1.00 ± 0.14 vs. 1.96 ± 0.38<br>t(6) = 2.39; F = 14.6<br>**p = 0.027*	1.00 ± 0.01 vs. 2.09 ± 0.40<br>t(5) = 2.70; F = 4635<br>**p = 0.021*
TJP2	1.00 ± 0.21 vs. 1.04 ± 0.15<br>t(4) = 0.17; F = 2.06<br>n.s. *p* = 0.438	1.00 ± 0.21 vs. 1.07 ± 0.07<br>t(2) = 0.30; F = 8.28<br>n.s. *p* = 0.395	1.00 ± 0.14 vs. 0.76 ± 0.05<br>t(3) = 1.57; F = 3.47<br>n.s. *p* = 0.115	1.00 ± 0.07 vs. 0.75 ± 0.05<br>t(4) = 2.92; F = 1.16<br>**p = 0.019*
TJP3	1.00 ± 0.10 vs. 1.15 ± 0.26<br>t(3) = 0.56; F = 7.32<br>n.s. *p* = 0.311	1.00 ± 0.10 vs. 1.08 ± 0.07<br>t(4) = 0.65; F = 1.64<br>n.s. *p* = 0.278	1.00 ± 0.22 vs. 0.73 ± 0.06<br>t(2) = 1.16; F = 3.84<br>n.s. *p* = 0.176	1.00 ± 0.07 vs. 0.78 ± 0.06<br>t(5) = 2.31; F = 1.75<br>**p = 0.033*
CLDN-7	1.00 ± 0.11 vs. 1.20<br>t(4) = 1.18; F = 1.56<br>n.s. *p* = 0.154	1.00 ± 0.11 vs. 1.15 ± 0.02<br>t(2) = 1.45; F = 30.8<br>n.s. *p* = 0.138	1.00 ± 0.11 vs. 0.75 ± 0.08<br>t(4) = 1.86; F = 1.01<br>n.s. *p* = 0.068	1.00 ± 0.02 vs. 0.78 ± 0.08<br>t(5) 2.70; F = 47.8<br>**p = 0.020*
CLDN-8	1.00 ± 0.18 vs. 0.75 ± 0.13<br>t(4) = 1.12; F = 1.99<br>n.s. *p* = 0.166	1.00 ± 0.18 vs. 0.50 ± 0.04<br>t(2) = 2.69; F = 23.7<br>*p = 0.053*	1.00 ± 0.17 vs. 0.80 ± 0.10<br>t(3) = 1.01; F = 1.50<br>n.s. *p* = 0.191	1.00 ± 0.07 vs. 1.20 ± 0.15<br>t(7) = 1.19; F = 7.95<br>n.s. *p* = 0.137
CLDN-12	1.00 ± 0.08 vs. 0.73 ± 0.21<br>t(3) = 1.26; F = 7.40<br>n.s. *p* = 0.156	1.00 ± 0.08 vs. 0.53 ± 0.05<br>t(3) = 5.25; F = 2.30<br>***p = 0.005*	1.00 ± 0.28 vs. 0.71 ± 0.13<br>t(3) = 0.91; F = 2.24<br>n.s. *p* = 0.215	1.00 ± 0.10 vs. 0.99 ± 0.19<br>t(7) = 0.06; F = 7.59<br>n.s. *p* = 0.475
CLDN-19	1.00 ± 0.01 vs. 0.83 ± 0.03<br>t(3) = 5.31; F = 4.93<br>***p = 0.008*	1.00 ± 0.01 vs. 0.91 ± 0.02<br>t(4) = 4.02; F = 1.83<br>***p = 0.009*	1.00 ± 0.04 vs. 1.80 ± 0.42<br>t(5) = 1.91; F = 277<br>n.s. *p* = 0.057	1.00 ± 0.02 vs. 1.64 ± 0.38<br>t(5) = 1.68; F = 739<br>n.s. *p* = 0.077
MAGL	1.00 ± 0.16 vs. 1.14 ± 0.16<br>t(4) = 0.63; F = 1.04<br>n.s. *p* = 0.282	1.00 ± 0.16 vs. 0.85 ± 0.02<br>t(2) = 0.92; F = 45.1<br>n.s. *p* = 0.226	1.00 ± 0.14 vs. 0.91 ± 0.06<br>t(3) = 0.62; F = 3.27<br>n.s. *p* = 0.292	1.00 ± 0.03 vs. 1.22 ± 0.08<br>t(6) = 2.68; F = 14.4<br>**p = 0.018*

DAGLBETA	1.00 ± 0.22 vs. 0.99 ± 0.16<br>t(2) = 0.03; F = 131<br>n.s. *p* = 0.490	1.00 ± 0.22 vs. 0.79 ± 0.04<br>t(2) = 0.93; F = 31.1<br>n.s. *p* = 0.222	1.00 ± 0.02 vs. 0.76 ± 0.06<br>t(6) = 3.91; F = 18.6<br>***p = 0.004*	1.00 ± 0.05 vs. 0.95 ± 0.08<br>t(7) = 0.55; F = 4.14<br>n.s. *p* = 0.299

Supplementary Table S4: Summary of the relative expression of the differentially expressed genes (DEGs) and the statistical analysis performed to identify these DEGs from the NanoString analysis. Unpaired one-tailed t-test with Welch's correction was performed to identify significant differences across conditions. Data presented includes t-ratios, degrees of freedom, F-values, and p-values; * = p<0.05, ** = p<0.01. SD = standard diet, WD = western diet, n.s. = not significant.
